# Creativity as an Opportunity to Stimulate a Cognitive Approach to Tourist Demand

**DOI:** 10.3389/fpsyg.2021.711930

**Published:** 2021-08-04

**Authors:** Tiago M. Lopes, Teresa Palrão, Rosa I. Rodrigues

**Affiliations:** ^1^Escola Superior de Hotelaria e Turismo do Estoril, Estoril, Portugal; ^2^Centro de Estudos Geográficos, Universidade de Lisboa, Lisbon, Portugal; ^3^Universidade Lusófona, Lisbon, Portugal; ^4^Instituto Superior de Gestão, Lisbon, Portugal

**Keywords:** creative tourism, cognitive psychology, tourism experience, tourist behavior, destination planning

In its genesis, tourism and hospitality are within the area of service provision, so they are largely influenced by human interactions and the behaviors resulting from them (Kandampully et al., 2018). Today, countless emotional, social, communicational, or interactive dimensions are added to technical services, to increase personalized and targeted staging of tourist services. These dimensions require, according to Qiu et al. (2018) the proper comprehension of tourism from a psychological point of view. This approach may help to understand, describe and explain the attitudes, perceptions and motivations of the tourists regarding the choice of a destination over another. Knowledge of visitors' preferences is essential to better meet their expectations when they encounter the culture, customs, or traditions of the host country (Fang, 2020).

Tourist behavior is based on social, emotional, motivational, and cognitive aspects. Visitors actively participate in the construction of their trips before, during and after the visit, requiring time, effort and money. They do it because the expected consequences of the process (e.g., novelty, enthusiasm, pleasure, prestige, socialization, learning or well-being) are valued by themselves or by people who are relevant to them (Prebensen et al., 2014). For this purpose, it seems urgent, in addition to purely behavioral or sociological issues about tourism demand, to understand and associate new paradigms of cognitive psychology and neuropsychology to tourism, to better anticipate emotional factors and to promote fair expectations (Scott, 2020).

Cognitive psychology studies the mental processes underlying behavior, namely, memory and the ability to store, transform and apply assimilated knowledge. It can thus be applied to better understand the transformation of traditional cultural tourism into creative supply that triggers authenticity, enriches the tourist experience, and encourages visitor involvement with local history (Bec et al., 2019). This approach, as suggested by Moyle et al. (2019), implies a strong association of the tourism sector (which is already a multidisciplinary discipline) to new and innovative research axes, combining social and natural sciences.

Destinations seek to deeply involve visitors in the main centralities of supply, framing a great diversity of novel stimuli (e.g., gastronomic, historical, environmental, community), to achieve their satisfaction, without neglecting the authenticity of the place (Coelho et al., 2018). Quality is assumed by destinations as an essential feature to ensure customer loyalty and competitiveness. However, it appears that the majority of tour operators are small and medium-sized companies. These companies show a low degree of qualification concerning the administration of their business (being focused on technical dimensions of the process) and, as such, managers are essentially focused on maintaining operational activity and not on developing future strategies with a focus on the client's behavioral patterns.

Following the view of Park et al. (2017), we believe that entrepreneurs have in creative tourism an opportunity to innovate. Regardless of the type of surrounding environment, well-being, nature, authenticity, experiential and creative experiences are at the heart of the relationship that is going to connect visitors and locals. Each tourist experience takes on a different meaning and it's largely influenced by memories, emotions, and the way the tourist interacts with the visited place (Wood and Kenyon, 2018). From the cognitive perception, as Skavronskaya et al. (2020) stated, creative tourism may stimulate effective novelty, associated with the encounter of different or usual stimuli at destinations, which will enhance tourist attention and endorse new emotional outputs and memories.

There were several approaches by researchers who sought to understand the concept of tourist experience from an early age, defining types and motivations of visitors, and exploring the importance of authenticity, suitability or perceived image of each tourist (Cohen, 1979; Otto and Ritchie, 1996). Pine and Gilmore (1999) shown that an experience occurs when a company intentionally involves individual customers in memorable activities. The tourist trip is commonly voluntary and undertaken with the desire to meet personal and family hedonic needs—not because tourists must, but because they want to, guided by their motivation, goals, interests, skills, or experience.

New dynamics of the tourism sector reveal an increasingly less passive and contemplative demand, which values and enjoys experiential (Coelho et al., 2018), emotional (Volo, 2021), captivating and engaging (Moscardo, 2020), transforming (Sheldon, 2020), smart (Xiang et al., 2021) and creative (Richards, 2019) features in the destinations.

By fostering competitiveness among destinations, the tourism sector fosters integrated planning and governance strategies and stimulates the hybridization of its tangible and intangible heritage (Della Lucia and Trunfio, 2018), while offering memorable (Kirillova et al., 2017), transformative (Teoh et al., 2021), co-creative (Campos et al., 2018) and significant experiences (Ross, 2020).

Creative tourism proves to be a transversal axis to the design of destinations, involving people, processes, products, and scenarios (Wang et al., 2020). As Richards (2020) states, from the elementary nature of some crafts and workshops, the concept of creative tourism has evolved in recent decades to new opportunities based on sharing and co-creation of knowledge. This approach associates producers, consumers, technology, talent and skills and generates new products, content and strong intangible experiences. Tourists are increasingly engrossed with local realities, actively participating and interacting with residents. Currently, creative tourism may even strategically guide tourism planning at local and regional levels.

We believe in the interest of crossing research in creative tourism and cognitive psychology, as it may lead to better adaptation of the created tourism environment. Although creative tourism has been strongly promoted in the cultural axes of the tourist supply, we consider that any scenario (e.g., natural, rural, city, coastal) may be enhanced with the dynamism of transformative experiences. This approach will certainly lead to a greater sense of proximity to the local culture, and to active participation in creative activities which strengthen appropriation and involvement with the visited territory.

Garcês et al. (2018) added that tourism, being a hedonic consumption, promotes happiness and well-being amongst visitors, and that well-being is a strong marketing tool. The tourism sector opens doors to new opportunities for healthy and sustainable living, making visitors feel motivated to visit places that convey positive emotions and authentic and memorable experiences (Skavronskaya et al., 2020). Majeed and Ramkissoon (2020) reinforce this idea, stressing that health and wellness tourism—particularly after a pandemic context such as COVID 19, are increasingly valued by visitors. These authors suggest that a combination of physical and built environments could condition human perception and interactions to induce a sense of healing, happiness, place attachment, satisfaction, and quality of life, which ultimately may lead to re-visitation (Ramkissoon, 2020).

The set of COVID-19 effects on tourism also justifies a quick understanding of its implications on various stakeholders, such as visitors, industry entrepreneurs, governments, communities, related sectors, among others. Collaboration and multidisciplinary research are crucial to encourage tourism activity while working toward the empowerment, reassurance, and confidence of the public and economic agents (Wen et al., 2020). These areas are highly dependent on cognitive dimensions of tourism experience and could be enhanced through creativity—or “the production of novelty” (Cropley, 1999, p. 253).

From these perspectives, we believe that a better comprehension of creative and cognitive stimuli among tourists may enhance and enrich cultural, natural, social, and personal resources of the tourism supply. The appropriateness of this approach is also defended as constituting a guiding vehicle for the strategic policies of destinations since it will allow the establishment of new relationships between the various niches that make up the offer, and holistically condition the satisfaction of visitors.

## Author Contributions

All authors listed have made a substantial, direct and intellectual contribution to the work, and approved it for publication.

## Conflict of Interest

The authors declare that the research was conducted in the absence of any commercial or financial relationships that could be construed as a potential conflict of interest.

## Publisher's Note

All claims expressed in this article are solely those of the authors and do not necessarily represent those of their affiliated organizations, or those of the publisher, the editors and the reviewers. Any product that may be evaluated in this article, or claim that may be made by its manufacturer, is not guaranteed or endorsed by the publisher.
